# The complicated role of venous drainage on the survival of arterialized venous flaps

**DOI:** 10.18632/oncotarget.14845

**Published:** 2017-01-27

**Authors:** Weidong Weng, Feng Zhang, Bin Zhao, Zhipeng Wu, Weiyang Gao, Zhijie Li, Hede Yan

**Affiliations:** ^1^ Department of Orthopedics (Division of Plastic and Hand Surgery), The Second Affiliated Hospital and Yuying Children's Hospital of Wenzhou Medical University, Wenzhou, China; ^2^ Division of Plastic Surgery, University of Mississippi Medical Center, Jackson, Mississippi, USA

**Keywords:** arterialized venous flap, venous drainage, venous congestion, survival mechanism

## Abstract

The arterialized venous flap (AVF) has been gradually popularized in clinical settings; however, its survival is still inconsistent and the role of venous drainage remains elusive. In this study, we aimed to investigate the role of venous drainage on the flap survival of arterialized venous flaps. An arterialized venous flap was outlined symmetrically in the rabbit abdomen. The arterial perfusion flap with a unilateral vascular pedicle was taken as the control group and three other experimental groups (I, II and III) were designed based on the number of drainage veins (*n* = 1, 2 and 3 in the three groups, respectively). Compared with the control group, significant venous congestion was noted in all the experimental groups and the most severe one was seen in group I; while no statistical difference was observed between groups II and III. Similar results regarding blood perfusion state, epidermal metabolite levels and flap survival status were obtained among the three groups. These findings suggested that venous drainage is vital in the survival of the flap, but unlike in the arterial perfusion flaps, the problem of venous congestion can only be partially solved by increasing the number of draining veins. Further studies are warranted to gain insight into this complicated issue.

## INTRODUCTION

With the increasing aesthetic demands in plastic and reconstructive surgeries, the development of thin flaps with high quality has gained great interest in recent years [1]. A variety of flaps have been designed and utilized in clinical practice for resurfacing different kinds of soft tissue defects; however, only a few flaps are well accepted in terms of aesthetic concerns [2, 3]. The arterialized venous flap (AVF) is one of the flaps that can meet the aesthetic needs in plastic and reconstructive surgeries based on the advantages of being non- bulky, ease of design and harvest, no limitation of the donor sites, and less donor-site morbidity [4]. AVFs have been highly popularized in the reconstruction of small skin defects in hand surgery and achieved satisfactory outcomes both functionally and aesthetically. Nonetheless, AVFs, as a non-physiological flap, still suffer from a high partial or even total flap loss rate in literature, especially for the reconstruction of relatively large defects [5, 6]. The philosophy of this phenomenon is still unclear.

The common manifestations of AVFs after surgery are swelling, venous congestion and blister formation [4], indicating the existence of a venous drainage problem. Therefore, anastomoses of larger or more veins to relieve venous congestion have been advocated and attempted, however this issue is yet to be solved completely [7, 8]. In this study, we aim to investigate the role of venous drainage on the survival of AVFs in rabbits, attempting to develop a possible strategy on survival improvement of this flap.

## RESULTS

Thrombosis at the anastomosis site was found in two animals (one in group I and the other one in group II) at the end of the experiment. Further on, one animal in group II developed a large hematoma under the flap. These animals were excluded from this study and replaced with three other animals. The patency of the anastomosis sites was confirmed in all other animals and all survived uneventfully with no obvious weight loss. All rabbits were euthanized with overdose of pentobarbital and pneumothorax at the end of experiment and this method of euthanasia in this study was specifically approved by our Institutional Animal Care and Use Committee of Wenzhou Medical University. The rabbits used for detection of water content and epidermal metabolite levels were sacrificed 3 days after sample collection postoperatively and all others were euthanized 14 days after surgery.

After surgery, the average blood perfusion unit of the flap in all three experimental groups was significantly lower than that of the control group (conventional arterial perfusion group) in all the observed time points (all *p* < 0.01) and the average value of the experimental group I was the lowest among the four groups, but there were no significant differences between the experimental groups II and III in all time points (all *p* > 0.05) (Table 1).

**Table 1:** Results of blood flow measurements after surgery^▲^

Groups	Day 1	Day 3	Day 5	Day 7
Control group (Arterial perfusion group)	57.00 ± 1.41	61.33 ± 0.81	62.00 ± 1.41	62.50 ± 1.87
Experimental group I (inflow:outflow,1:1)	27.83 ± 1.17	33.50 ± 1.05	33.00 ± 0.89	33.17 ± 4.87
Experimental group II (inflow:outflow,1:2)	39.33 ± 1.37	44.50 ± 1.87	42.83 ± 0.98	42.00 ± 1.09
Experimental group III (inflow:outflow,1:3)	38.83 ± 1.72	44.83 ± 1.60	44.17 ± 1.47	43.50 ± 1.38

^▲^Of all the observed time points, the blood perfusion units of the control group were all significantly different from the three experimental groups (all *p* < 0.001) and those of the experimental group I were also significantly different from the other two experimental groups (all *p* < 0.01); however, no significant differences were noted between the experimental group II and III (all *p* > 0.05).

**Figure 1:**
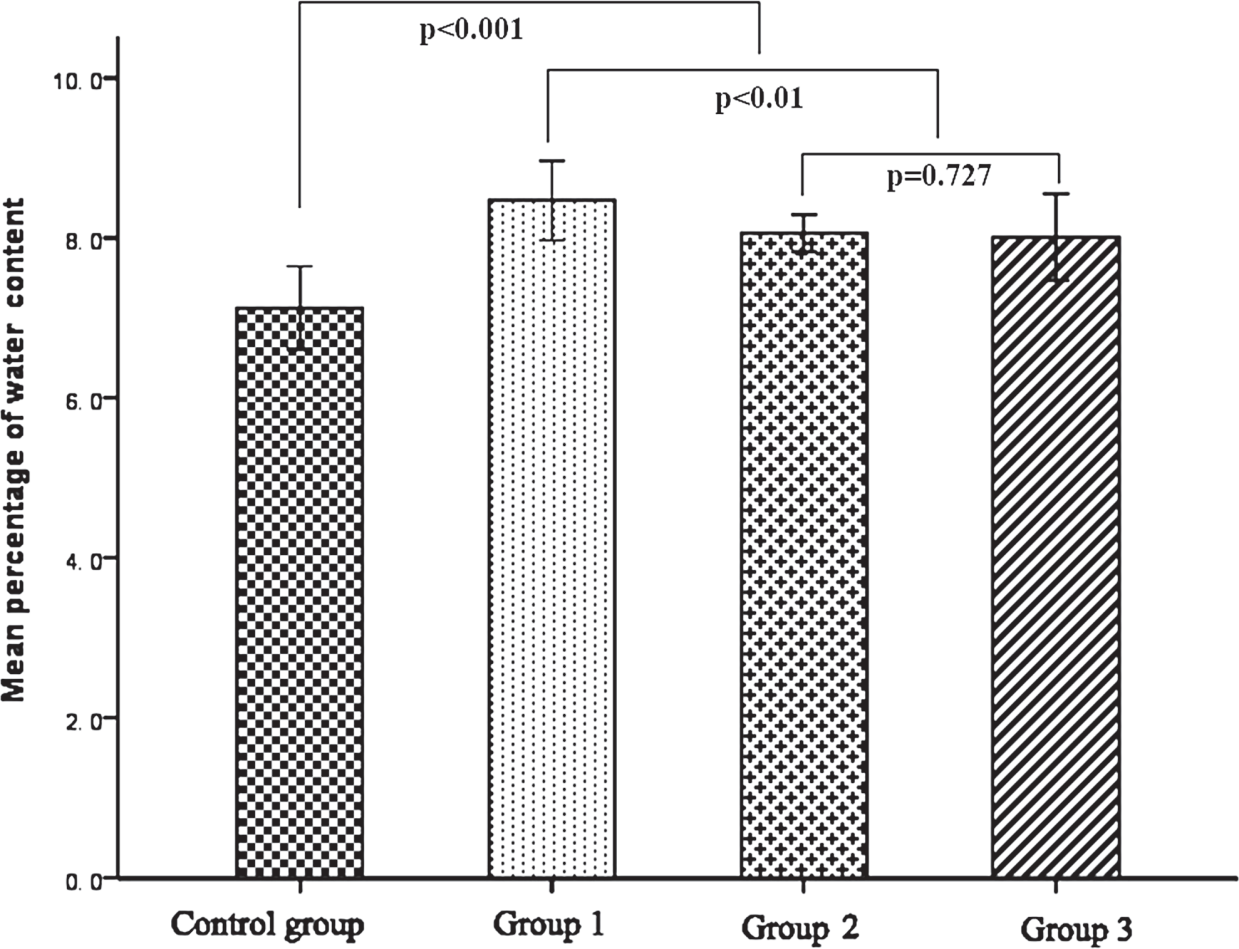
Results of water content 72 h after operation

In comparison with the conventional flap of the control group, the average values of water content in the experimental groups were all significantly higher than that of the control group (all *p* < 0.01); of the three experimental groups, the experimental group I showed a relatively higher value of water content when compared with the other two groups, while there were no significant differences between experimental groups II and III (*p* = 0.727) (Figure 1).

The results of the epidermal contents of glucose and lactate in each group were listed in Table 2. Significant differences in lactate and glucose levels were observed among these four groups (*p* < 0.01); while Mann-Whitney test with Bonferroni correction showed no significant differences in the level of glucose (*p* = 0.377) or lactate (*p* = 0.371) between experimental groups II and III, respectively; however, significantly higher levels of lactate and lower levels of glucose were noted in experimental group I in comparison with the other two experimental groups (all *p* < 0.01).

**Table 2:** Epidermal metabolite levels 72 hours after surgery

Groups	Glucose (mmol/gprot)	Lactate (mmol/gprot)	Lactate/Glucose Ratio
Control group^▲^ (Arterial perfusion group)	0.858 ± 0.028	1.130 ± 0.078	1.32 ± 0.09
Experimental group I^▼^ (inflow:outflow,1:1)	0.436 ± 0.025	1.710 ± 0.09	3.93 ± 0.31
Experimental group II^♥^ (inflow:outflow,1:2)	0.611 ± 0.016	1.539 ± 0.104	2.52 ± 0.18
Experimental group III (inflow:outflow,1:3)	0.623 ± 0.019	1.486 ± 0.119	2.38 ± 0.13

^▲^Values of the control group were significantly different from the three experimental groups (all *p* < 0.01); ^▼^ those of the experimental group I were also significantly different from the other three groups (all *p* < 0.01); ^♥^ no differences were noted between the experimental group II and III (Glucose: *p* = 0.377; Lactate: *p* = 0.371).

**Figure 2:**
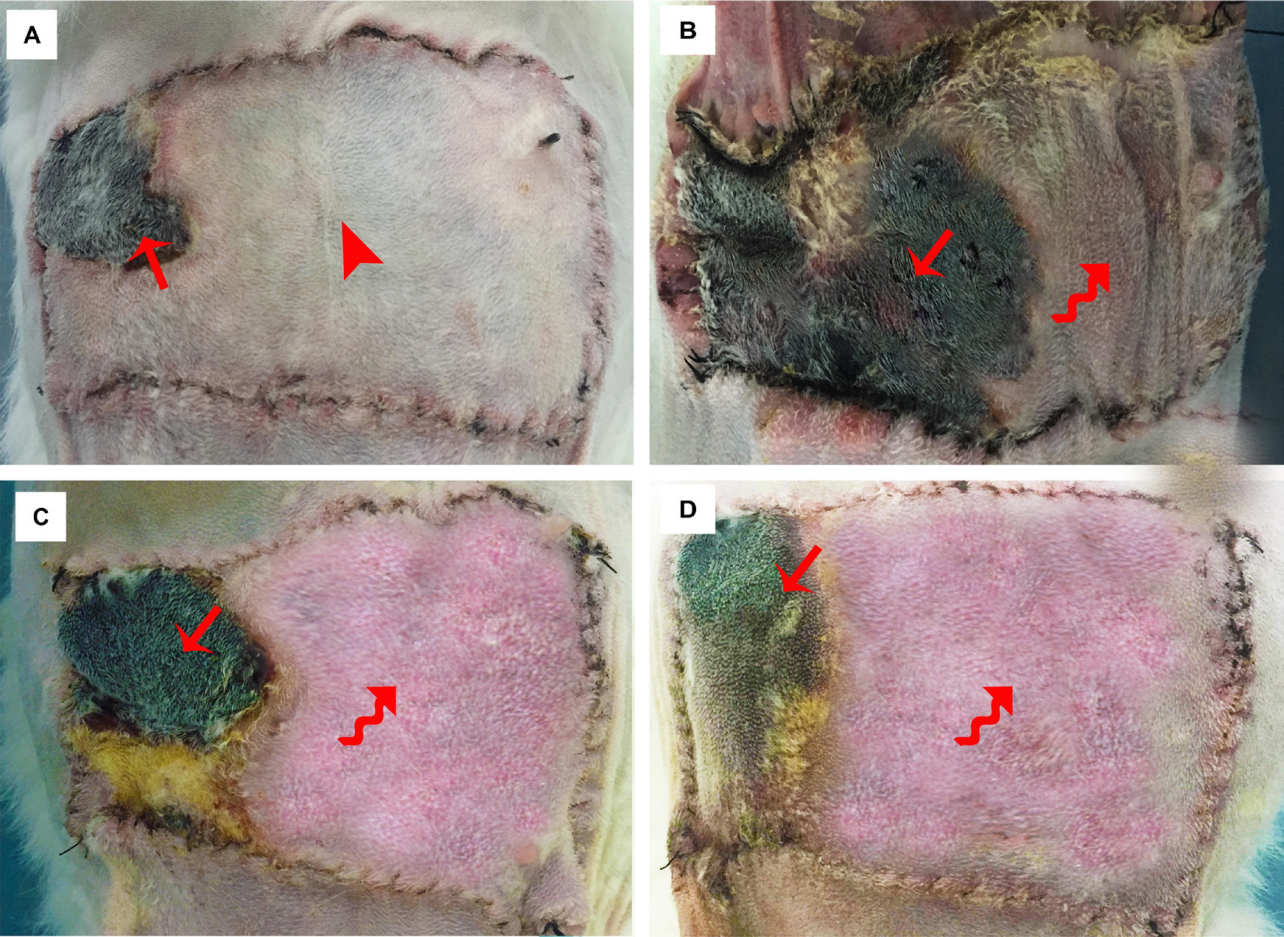
Flap survival status 14 days after surgery (**A**) Control group: a small area of flap loss presented around the contralateral corner of the flap with no obvious swelling or venous congestion.(The arrow head shows the area without obvious swelling and venous congestion and the straight arrow indicates the small necrosed area). (**B**) Experimental group I: more than half of the flap was necrosed with swelling and venous congestion around the necrosed area. (The curved arrow shows the area with obvious swelling and venous congestion and the straight arrow indicates the necrosed area). (**C**) Experimental group II: partial flap loss was noted in the distal part of the flap with swelling and venous congestion around the necrosed area. (The curved arrow shows the area with significant swelling and venous congestion and the straight arrow indicates the necrosed area). (**D**) Experimental group III: partial flap loss was noted in the distal part of the flap with swelling and venous congestion around the necrosed area. (The curved arrow shows the area with significant swelling and venous congestion and the straight arrow indicates the necrosed area)

**Figure 3:**
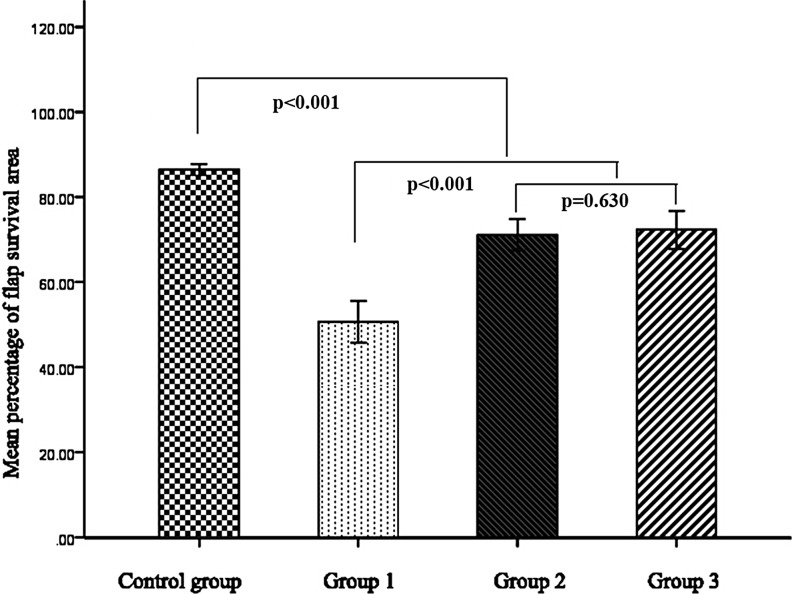
Results of mean percentage of flap survival area

The flap survival status is shown in Figure 2. In the control group, no obvious swelling or venous congestion was noted postoperatively, instead, the distal part of the flap presented low perfusion signs of paleness and became more significant with time passing by and gradually resulting in partial flap loss around the contralateral corner of the flap 14 days after surgery(Figure 2A). In contrast, the flaps in the three experimental groups all presented with venous congestion and swelling shortly after surgery and this condition became more obvious three days after operation, and then subsided gradually, resulting in partial flap loss. This phenomenon in the experimental group I was more evident when comparing with the other two experimental groups. Accordingly, partial flap loss was observed in the three experimental groups at day 14 post operation (Figure 2C–2D), of which relatively better flap survival status was found in the experimental groups II and III. Statistically, significant differences in the percentage of flap survival area was noted among the four groups and the experimental group I had the lowest percentage of flap survival area in these groups (all *p* < 0.001); however, no significant differences were found between experimental groups II and III (*p* = 0.630) (Figure 3).

## DISCUSSION

There have been considerable controversies about the AVF from the beginning of its advent. This flap differs from the traditional flap because it is perfused only through the venous system [9, 10]. Several survival mechanisms regarding this kind of flaps, such as the theories of “A-V shunting”[11],“reverse flow”[12] and “capillary bypass”[13], have been brought forward, yet no consensus on its exact mechanism has been comprehensively addressed. The critical problem related to its survival is the challenge of its unsolved over-perfusion state, which is believed to be responsible for partial or even total flap loss [14].

In literature, several approaches have been attempted to solve the problem of venous congestion and improved the survival status of AVFs, such as using a relatively larger efferent vein and a relatively smaller afferent vein [15], adopting surgical or chemical delay tactics [16, 17], and anastomosing multiple draining veins [18]. Woo SH et al. [18]proposed that more than two draining veins should be anastomosed for a better flap survival in the application of AVFs. However, due to the complexity of venous networks in selected AVFs, such as the follow-through type, V-shape type, and H-shape type, etc., the correlation between the number of draining veins and the status of flap survivals has not been well described. In this study, the status of venous congestion was partially relieved by increasing the number of veins for venous drainage. In comparison with the experimental group I (inflow: outflow, 1:1), flaps in the experimental groups II(inflow: outflow, 1:2) and III (inflow: outflow, 1:3) showed significant decreased levels of water content and relatively better blood perfusion and improved epidermal metabolite levels in terms of glucose and lactate, contributing to better flap survival status. However, there were no significant differences in all the observed parameters between the experimental groups II and III, indicating that the relief of venous congestion seems not to be simply and positively related to the numbers of draining veins. Therefore, increasing the number of draining veins can only partially benefit the survival of AVFs and there must be some deep reasons for this phenomenon.

It is a commonsense that the problem of venous congestion in physiological flaps can be naturally settled down by increasing the number of draining veins [19–21]. The reasons why this strategy does not work efficiently for AVFs still remain elusive. In our previous study [10], we found that consistent venous congestion occurred in the peripheral area in a flow-through type of AVF and its hemodynamic feature is characterized as an integrated perfusion mode. Under this perfusion mode, the pressure in the central arterialized vein was high and the pressure difference between the two ends was subtle. This situation prevented blood return from the peripheral area, resulting in an intractable over-perfusion state. To some extent, the problem of venous congestion may be resolved by increasing the number of draining veins in this follow-through type of AVF, but it could not be completely solved due to its non-physiological microcirculation status and it became even more complicated in other types of AVFs (for instance, the Y- or H-type AVF) [7]. Further investigations are needed to explore the mechanism of venous congestion in AVFs so as to eventually develop new strategies in dealing with this problem.

In this experiment, the ipsilateral thoracoepigastric vein (TEV) was kept intact for venous drainage in group I. Since there are differences in venous networks in the rabbit abdomen, disparities in results might occur if the contralateral TEV (distal or proximal) was selected as the draining vein for group I. However, the current experimental design is competent in terms of the purpose of this study. Further studies are warranted to investigate the influence of distribution of draining veins in flap survival of AVFs.

In conclusion, venous drainage is vital in the survival of AVFs and this problem can only be partially improved by increasing the number of draining veins. Further studies are warranted to gain insight into this complicated issue.

## MATERIALS AND METHODS

### Animal models and grouping

As described in detail in our previous study [1, 10], adult white Japanese rabbits of both sexes, weighing between 3.5 and 4 kg, were used. All experiments were approved by the Institutional Animal Care and Use Committee of Wenzhou Medical University and carried out according to the ethical approval and the National Research Council's guidelines for the care and use of laboratory animals. All animals were anesthetized with pentobarbital (90 to 150 mg plus 30 to 50 mg h1 IV; Sigma).After the rabbit abdominal area was shaved, the flap was then designed on it. The rabbits were kept under anesthesia by re-injection of pentobarbital during the operation.

**Figure 4:**
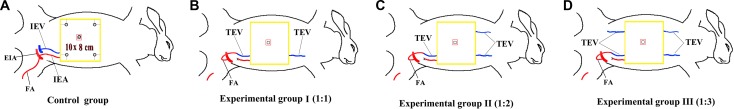
Animal model and grouping A 10 × 8 cm (length x width) skin flap was designed symmetrically along the middle line of the abdomen (**A**) Control group: single side of inferior epigastric artery and vein was preserved as a physiological flap. The circles including four ones located in 1.5 cm away from both sides and one in the middle indicate the location for blood flow measurement and the black square in the center area shows the specimen harvested for water content study and the area between the red square and black squared is harvested for metabolite analysis.(IEV, inferior epigastric vein; IEA, inferior epigastric artery; EIA, external iliac artery; FA, femoral artery.) (**B**) Experimental group I: one side of femoral artery was freed and transferred reversely to anastomose with the proximal end of the thoracoepigastric vein, simply leaving the distal thoracoepigastric vein of this side intact to provide venous outflow and all other arteries and veins connected with the flap were ligated.(TEV, thoracoepigastic vein; FA, femoral artery). (**C**) Experimental group II: similar to experimental group I, but additionally leaving the contralateral distal side of thoracoepigastric vein intact. (TEV, thoracoepigastic vein; FA, femoral artery). (**D**) Experimental group III: similar to experimental group II, but additionally leaving the contralateral proximal side of thoracoepigastric vein intact. (TEV, thoracoepigastic vein; FA, femoral artery).

Forty-eight rabbits were used and randomly divided into four groups (*n* = 12). The animal model was modified based on our previous study [1, 10]. In brief, a 10 × 8 cm (length x width) skin flap was designed symmetrically along the middle line of the abdomen; the flap was tailored including the epidermis, dermis, subcutaneous tissue, and the panniculus carnosis. In the control group (conventional arterial perfusion group), the flap was dissected, leaving only one side of the inferior epigastric artery and vein intact as a physiological flap; in experimental group I (inflow: outflow, 1:1), one side of femoral artery was freed and transferred reversely to anastomose with the proximal end of the thoracoepigastric vein using 11–0 sutures, simply leaving the distal thoracoepigastric vein of this side intact to provide venous outflow and all other arteries and veins connected with the flap were ligated; in experimental group II (inflow: outflow, 1:2), similar to experimental group I, but additionally leaving the contralateral distal side of thoracoepigastric vein intact; in experimental group III (inflow: outflow, 1:3), similar to experimental group II, but additionally leaving the contralateral proximal side of thoracoepigastric vein intact (Figure 4A–4D). The flaps in each group were then sutured back *in situ* after handling the vascular bundles.

All surgical procedures were performed aseptically. In order to guard against pressure and friction, a soft padded dressing was applied to cover the flap. In addition, a special collar was also utilized to prevent the wound and dressing from being chewed during the course of the experiment. Antibiotics and analgesia were administered for three days postoperatively. Every rabbit was kept in a separated clean cage and could access the food and water ad libitum. All animals were monitored every day, focusing on the general condition, appetite, body weight and fur. Dressings were changed every two days or timely whenever contaminated or loosened.

### Blood flow measurement

Tissue blood perfusion in flaps was measured on randomly selected 6 animals in each group using laser Doppler flowmetry (Peri-Flux system 5000; Perimed, Jarfalla, Sweden) on 1, 3, 5 and 7 days postoperatively. Measurement was carried out on five points of the flap: four corners and the median portion of the flap (Figure 4A). The room temperature was maintained at around 20°C during the measurement. For consistency, three measurements were obtained and each measurement lasted at least 30 seconds. The average value from the five points was used as the final parameter for evaluation. The results were expressed as blood perfusion unit.

### Water content measurement in flaps

Six rabbits were randomly selected in each group 3 days after surgery. As described in detail in our previous study [10], the flap samples (1 × 1 cm^2^) were harvested in the center of the flap (Figure 1, the black square) and dried with a stencil for analysis and the wet weight of the sample was obtained by a precision electronic scale. The flap was then placed in a drying oven and maintained for 24 hours at 65°C and the dry weight of the flap was measured with the same precision scale. The water content is calculated using the following formula [22]: Water content (%) = (Wet weight−Dry weight)/Wet weight × 100

### Detection of epidermal metabolite levels

The epidermal metabolite levels of glucose and lactate were measured 72 hours after operation. The same six rabbits selected for water content analysis in each group were used. As described in detail in our previous study [1, 10], while the rabbits were still alive and anesthetized, specimens of 1.5 × 1.5 cm in size (after harvesting the specimen of 1 × 1 cm square for water content analysis in the middle) were harvested in the center of each flap (Figure 4). The specimens were then frozen in liquid nitrogen immediately and grinded into 10% tissue homogenate. The total protein content of the tissue lysate samples was measured using the Bradford assay [23]. The contents of glucose and lactate were determined by colorimetric assay kits (Nanjing Jiancheng Bioengineering Institute, Jiangsu, China) based on the following equation: content of lactate or glucose(mmol / gprot) = [(OD of the sample tube - OD of blank tube)/(OD of standard tube-OD of standard blank tube)] × concentration of standard sample / total protein content [1].

### Viability assessment

Flap viability was visually evaluated on postoperative days 1, 3, 5, 7, 11 and 14.Viability was assessed by flap color, degree of swelling, texture, hair growth, and the presence of necrosis. Objective measurement of flap survival was performed 14 days postoperatively. The survival areas of the flap were calculated on those without evidence of necrosis and then marked on a template. Using an M2 Image Analysis System (Imaging Research Inc, St. Catharines, Ontario, Canada), the template was photographed by a high-resolution video camera and input into a computer [1]. Quantification of flap survival was expressed as a percentage of the total flap surface area. The same investigator evaluated the flaps to minimize inter observer bias and variability.

### Statistical analysis

Statistical analysis was carried out using nonparametric method of Kruskal Wallis test with SPSS v. 19.0. The comparison between groups was performed with Mann-Whiteney *U* test with Bonferroni correction. Data were expressed as mean ± standard deviations.
